# Validation of MRC Centre MRI calf muscle fat fraction protocol as an outcome measure in CMT1A

**DOI:** 10.1212/WNL.0000000000006214

**Published:** 2018-09-18

**Authors:** Jasper M. Morrow, Matthew R.B. Evans, Tiffany Grider, Christopher D.J. Sinclair, Daniel Thedens, Sachit Shah, Tarek A. Yousry, Michael G. Hanna, Peggy Nopoulos, John S. Thornton, Michael E. Shy, Mary M. Reilly

**Affiliations:** From the MRC Centre for Neuromuscular Diseases (J.M.M., M.R.B.E., C.D.J.S., T.A.Y., M.G.H., J.S.T., M.M.R.) and Neuroradiological Academic Unit (S.S.), UCL Institute of Neurology, London, UK; Carver College of Medicine (T.G., P.N., M.E.S.) and Department of Radiology (D.T.), University of Iowa, Iowa City.

## Abstract

**Objective:**

To translate the quantitative MRC Centre MRI protocol in Charcot-Marie-Tooth disease type 1A (CMT1A) to a second site; validate its responsiveness in an independent cohort; and test the benefit of participant stratification to increase outcome measure responsiveness.

**Methods:**

Three healthy volunteers were scanned for intersite standardization. For the longitudinal patient study, 11 patients with CMT1A were recruited with 10 patients rescanned at a 12-month interval. Three-point Dixon MRI of leg muscles was performed to generate fat fraction (FF) maps, transferred to a central site for quality control and analysis. Clinical data collected included CMT Neuropathy Score.

**Results:**

Test-retest reliability of FF within individual healthy calf muscles at the remote site was excellent: intraclass correlation coefficient 0.79, limits of agreement −0.67 to +0.85 %FF. In patients, mean calf muscle FF was 21.0% and correlated strongly with disease severity and age. Calf muscle FF significantly increased over 12 months (+1.8 ± 1.7 %FF, *p* = 0.009). Patients with baseline FF >10% showed a 12-month FF increase of 2.9% ± 1.3% (standardized response mean = 2.19).

**Conclusions:**

We have validated calf muscle FF as an outcome measure in an independent cohort of patients with CMT1A. Responsiveness is significantly improved by enrolling a stratified patient cohort with baseline calf FF >10%.

Charcot-Marie-Tooth disease (CMT) or hereditary motor and sensory neuropathy is a common disease with a prevalence of 1 in 2,500. CMT causes progressive distal weakness, sensory loss, and disability, with CMT type 1A (CMT1A) accounting for more than half of all cases.^1^ There is currently no disease-modifying therapy although a number of promising agents are in development. Trials of vitamin C were negative^2,3^ but highlighted the need for responsive outcome measures.

Quantitative skeletal muscle MRI has been widely studied as an outcome measure in muscle diseases,^4,5^ and we recently reported high responsiveness over 12 months in CMT1A in a UK cohort.^6^ Intramuscular fat accumulation occurs as a common feature of chronic pathology in muscle whether the primary pathogenesis is neurogenic or myopathic, and can be quantified with MRI using fat-water separation methods such as the 3-point Dixon technique.^5^ In our previous study, this provided the most responsive outcome measure with a 12-month total calf muscle fat fraction (FF) increase of 1.2% ± 1.5% (*p* = 0.008) and standardized response mean (SRM) of 0.83, far exceeding that of any clinical outcome measure.^6^ However, it was recognized that this required further validation in an independent CMT1A cohort before application in clinical trials.

We aimed to translate our MRC Centre MRI protocol to a second site for validation in an independent patient cohort. We also tested the potential of stratification based on baseline muscle FF to increase outcome measure responsiveness.

## Methods

MRI was performed at the University of Iowa, Iowa City, with anonymized MRI data transferred for analysis at the MRC Centre for Neuromuscular Diseases, UCL Institute of Neurology, London, UK.

Eleven patients with genetically confirmed CMT1A were recruited for examination at baseline and 12 months. Age, disease duration, and the CMT Neuropathy Score (CMTNS) (a composite disease severity scale ranging from 0 = normal to 36 = maximum severity) were recorded. For longitudinal comparisons, the examination score (CMTES) component was used. The Rasch-modified CMTNS and CMTES were calculated.^7^

**Figure 1:**
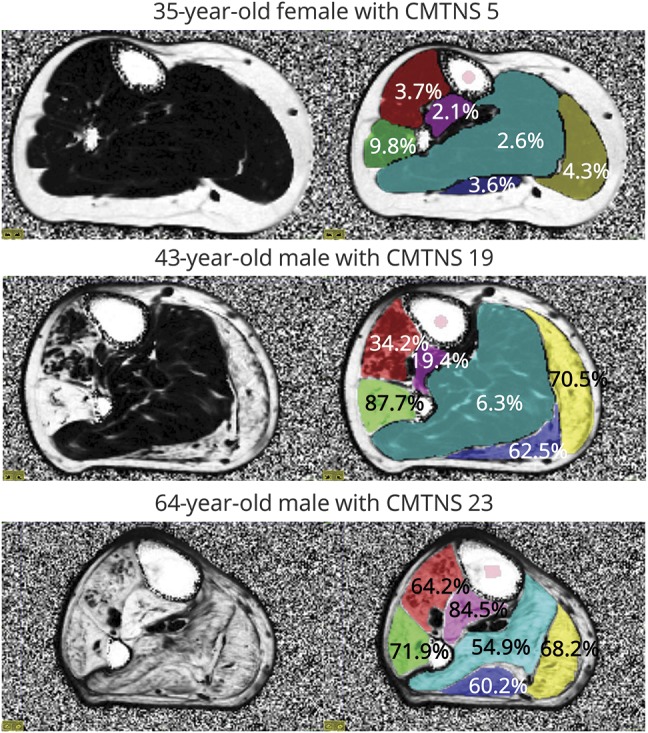
Quantitative MRI in a range of disease severity in Charcot-Marie-Tooth disease type 1A Axial fat fraction map of the right calf 130 mm distal to the lateral tibial plateau is shown on the left. Regions of interest (red: tibialis anterior/extensor hallucis longus; green: peroneus longus; purple: tibialis posterior; light blue: soleus; dark blue: lateral gastrocnemius; yellow: medial gastrocnemius) and respective mean fat fraction values are overlaid on the right. In the top patient, there is a slight increase in fat fraction in peroneus longus only. In the middle row, marked involvement is seen with endstage involvement of peroneus longus and both heads of the gastrocnemius. In the bottom row, there is severe involvement of all muscle groups. CMTNS = Charcot-Marie-Tooth Neuropathy Score.

The MRC Centre MRI protocol, identical to that used previously,^4^ was performed using a similar scanner (Siemens 3T TIM TRIO; Siemens Medical Solutions, Malvern, PA). In brief, calf-level 3-point Dixon MRI was performed centered 130 mm distal from the knee joint.^8^ Muscle-group cross-sectional regions of interest (figure 1) were drawn by a single observer (J.M.M.) on the right calf muscle on the central slice of the 3.45-millisecond Dixon acquisition to allow extraction of the corresponding combined all-muscle-group mean FF.

For remote-site quality-control qualification, 3 healthy volunteers were scanned with 2 undergoing repeat scanning on a different day to assess test-retest reliability, referenced to similar measurements performed in London.^9,10^

Statistical analysis was performed using SPSS Statistics 20.0 (IBM Corp., Armonk, NY). Group data were compared using student 2-tailed *t* tests, longitudinal change was assessed using paired *t* test, and MRI-clinical associations were assessed using Spearman rank correlation. Outcome measure responsiveness was assessed using the SRM (mean change divided by the change SD).

To investigate stratification, the remote-site data were combined with that from our previous study^4^ in a clinically similar CMT1A patient group (20 patients with CMT1A, 11 men, mean age 42.8 years, mean CMTES 8.0). Twelve-month FF changes and SRMs were calculated for patients with baseline overall muscle FFs above and below a threshold of 10%, for both sites separately and combined.

### Data availability

Anonymized data will be shared by request from any qualified investigator.

### Standard protocol approvals, registrations, and patient consents

Both sites' ethical review boards approved the study and all participants gave written informed consent.

## Results

Of the 11 patients with CMT1A assessed at baseline (6 women, 5 men, mean age 41, range 24–64 years; mean baseline CMTNS 15.2, SD 5.9, range 5–23), 10 underwent repeat assessment at median interval 1.03 years.

The remote-site qualification FF measurements showed excellent test-retest reliability (intraclass correlation coefficient 0.79; Bland-Altman limits of agreement −0.67 to +0.85 %FF). No images were affected by artifact, and repeat-scanning block positions were sufficiently consistent that the same numerical slice could be used for follow-up analysis in all cases.

Figure 1 shows FF in participants with mild, moderate, and severe CMTNS. The patients' baseline mean overall calf muscle FF was 21% (median 8.7, interquartile range 5.2–34.1). Peroneus longus had the highest mean FF (37.9%), with tibialis posterior the lowest (15.0%). Overall calf muscle FF correlated strongly with age (ρ = 0.71, *p* = 0.01), CMTNS (ρ = 0.695, *p* = 0.03), and Rasch-modified CMTNS (ρ = 0.81, *p* = 0.004).

**Figure 2:**
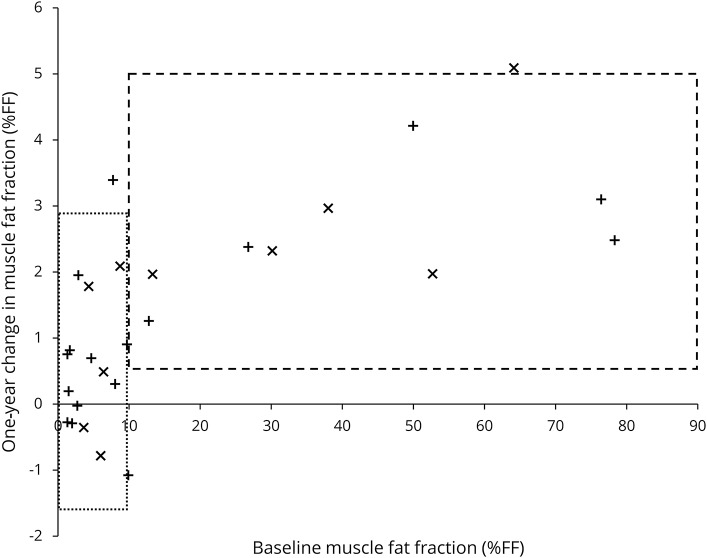
Absolute 1-year change in calf muscle fat fraction by baseline fat fraction x: US patients (this study); +: UK patients.^6^ Boxes are 95% confidence intervals for the pooled data grouped by baseline calf muscle fat fraction greater than or less than 10%. Fat accumulation is greater when baseline fat fraction is higher than 10%, which is consistent across the 2 studies (table).

Overall calf muscle FF increased over 12 months (+1.8 ± 1.7 %FF, *p* = 0.009), with increases in peroneus longus and lateral gastrocnemius also significant (table). Calf muscle FF showed high responsiveness (SRM 1.04), which improved on stratification: patients with baseline FF >10% showed a 12-month increase of 2.9 ± 1.3 %FF (SRM = 2.19) (table, figure 2). Compared with the subgroup with <10% calf muscle FF, those with FF >10% were older and more severely affected (mean age 50 years vs 34 years; mean Rasch-modified CMTES 18 vs 7). The Rasch-modified CMTES increased over 12 months but this was not statistically significant (+1.4 ± 2.6, *p* = 0.13).

**Table:**
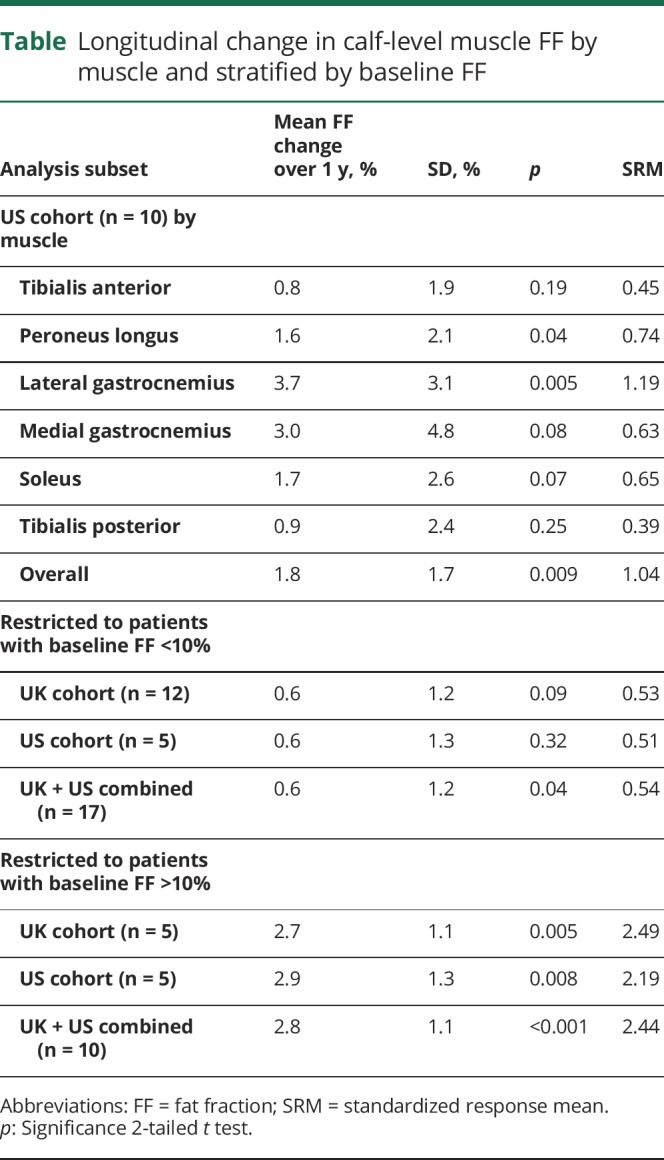
Longitudinal change in calf-level muscle FF by muscle and stratified by baseline FF

## Discussion

In this study, we successfully translated our calf-muscle MRC Centre MRI protocol to an international remote site, and further validated the value of calf-level muscle fat-fraction measurement in an independent cohort of CMT1A patients with high reliability, strong baseline cross-sectional clinical correlations, and high responsiveness. Stratification of the cohort based on baseline FF further improved the SRM to 2.44 in the combined cohort, resulting in an estimated sample size by the Lehr formula^8^ of 11 in each study arm of a randomized controlled trial with 0.8 power to detect a 50% slowing of disease progression at *p* < 0.05.

These data from an American cohort validate the results obtained in our original British CMT1A cohort^4^ (figure 2) and demonstrate international multisite trial readiness of quantitative lower limb MRI in patients with CMT1A. Utilization of a positioning protocol based on a fixed distance from the knee joint^6^ resulted in improved block positioning (10/10 scans within 1 cm of baseline in z-axis US vs 17/57 UK), confirming the superiority of this method. The 3-point Dixon method is robust after a brief study setup phase with no regions of interest excluded from analysis because of artifact in this study. The study setup phase was possible without an in-person site visit with images transferred internationally for quality-control purposes.

Outcome measure criterion validity was demonstrated with both cohorts showing similar FF correlations with disease severity (ρ = 0.81, *p* = 0.004 US with Rash-CMTS; ρ = 0.63, *p* = 0.003 UK with CMTES) and age (ρ = 0.84, *p* < 0.001 UK; ρ = 0.71, *p* = 0.01 US). Of note, the primary calf muscle FF outcome measure proposed previously^6^ showed similar increases (+1.2 ± 1.5 %FF UK, +1.8 ± 1.7 %FF US).

The SRM can be improved by participant stratification (figure 2): including only patients with baseline calf muscle FF >10% yielded an SRM >2 consistently in the United States, United Kingdom, and combined cohorts (table), markedly higher than the nonstratified groups. The inverse square relationship of SRM to sample size implies a potential dramatic reduction resulting in patient numbers needed in an equivalently powered study from 83 to 11 in each arm. Researchers would need to consider a potential ceiling effect in the trial design as the calf muscle FF approached the maximum level with 78% the highest value obtained across the 2 studies. There was less responsiveness in those with low baseline FF at calf level. Further studies to examine responsiveness of FF assessed in the distal calf or foot musculature are in process to identify a more responsive biomarker in this younger, more mildly affected subgroup. However, MRI calf muscle FF provides a highly responsive outcome measure in an unselected adult CMT population.

We have confirmed the reliability, validity, and responsiveness of the MRC Centre MRI quantified calf muscle FF protocol as an outcome measure in an independent cohort of patients with CMT1A. Selection of study participants with increased baseline calf muscle FF provides a highly responsive biomarker in this patient group, suitable for utilization in multicenter international clinical trials.

## Glossary

CMT: Charcot-Marie-Tooth disease
CMT1A: Charcot-Marie-Tooth disease type 1A
CMTES: Charcot-Marie-Tooth examination score
CMTNS: Charcot-Marie-Tooth Neuropathy Score
FF: fat fraction
SRM: standardized response mean
